# Scientization Under Pressure—The Problematic Role of Expert Bodies During the Handling of the COVID-19 Pandemic

**DOI:** 10.1007/s11115-022-00605-0

**Published:** 2022-04-21

**Authors:** Tom Christensen, Per Lægreid

**Affiliations:** 1 University of Oslo, Oslo, Norway; 2 University of Bergen, Bergen, Norway

**Keywords:** Scientization, Experts, COVID-19, Norway, politics, Administration

## Abstract

This article focuses on the role of experts in the Norwegian decision-making process in central government during the crisis management of the COVID-19 pandemic. It is based on a structural-instrumental and a cultural perspective. The main findings are that managing the pandemic led to a centralization of power in the hands of the political leadership, a blurring of the dichotomy between politics and administration, and a variety of expert advice. The crisis management also reflected the cultural appropriateness of a collaborative decision-making style, but it was not characterized by a scientization of policymaking. Rather than policymaking by experts it was policymaking informed by experts.

## Introduction

Most scholars concede that there is a grey zone between politics and administration; as well as implementing policy administrators also participate in advising political executives on policy development (Alford et al., 2017; Demir & Nynan, 2008; March & Olsen, 1989; Svara 2008; Waldo 1965). In practice, the public administration apparatus is a major political actor and not simply a neutral managerial tool. The organization of the administration is therefore a politically powerful instrument able to influence the content of public policy (Egeberg &Trondal, 2018). Moreover, the capacity and competence of the public management apparatus are crucial for the implementation of public policy. Thus, in reality public administration is comprised of political-administrative organizations that affect both the content and the implementation of public policy (Olsen, 2010).

One of the most important features of the administrative state is the quality of its civil servants’ expertise—in other words, public decisions should be based on professional decision-making premises, even if it is up to the political and administrative leadership to balance these with other premises (Rothstein & Teorell, 2008; Fukuyama, 2013; Raadschelders, 2017). There is a growing debate about the increasing reliance on scientific expertise in policymaking (Fischer, 2009). Some scholars use the term scientization of policy advice to refer to an increased reliance on advice based on scientific knowledge and provided by experts. This implies that scientized public agencies are endowed with scientific authority and framed in apolitical terms (Marcussen, 2006; Drori & Meyer, 2006; Christensen, 2018). It is often taken for granted that public administration experts represent a homogeneous body, implying that the advice the political leadership receives is unambiguous. In practice, however, that advice may differ because experts disagree or because evidence-based mechanisms are insecure and disputed. Adding to this, the organizational position of the various experts differs and hence also their impact on the decisions of executive leaders.

The handling of COVID-19 in various countries seems to be a good illustration of the differentiated impact of experts on public decision-making in crisis situations (Boin et al., 2020; Cairney & Wellstead, 2020). The COVID-19 crisis has tested the limits of what public bureaucracies are organized to handle. It is a transboundary and creeping mega-crisis characterized by complexity, uncertainty regarding means-end relations, and ambiguity regarding values. What is more, important decisions have had to be taken under great time pressure (Boin et al., 2020). Both governance capacity and governance legitimacy have been challenged (Christensen et al., 2016). Strategic crisis management is a managerial and functional matter requiring expert knowledge, but also a political issue involving value considerations and support from political executives as well as from citizens. Difficult trade-offs between life and death on the one hand, and individual freedom and civil rights on the other, together with economic issues related to unemployment and bankruptcy, social disruptions, and challenges for vulnerable groups, all need input from both types of experts if they are to be handled properly (Boin & ‘t Hart, 2003).

This case study focuses on the role of experts in the Norwegian decision-making process at the central level during the pandemic. The following research questions are asked:

- What are some of the main issues on which experts and the political leadership disagree in the handling of COVID-19 and is there a pattern in this disagreement?
- Do expert bodies agree in their recommendations to the government?
- What are some of the main explanations for why the experts have differing views and hence a differing influence on the final regulatory decisions of the government?

These questions are answered using public documents showing the advice given by the main specialized epidemiological body—the Norwegian Institute of Public Health (NIPH)—and by the more generalized health expert body—the Norwegian Directorate of Health (NDH). The latter has a coordinating role in health-related crises like pandemics and is organizationally closer to the Ministry of Health and Care Services (MH) than the NIPH. It is shown show how the MH and the government have balanced this often different advice in their regulations. Since the national regulatory rules are very complex and have changed more than 200 times during the pandemic, the focus is on some of the major issues involved in balancing the main considerations. Two perspectives taken from organization theory—structural and cultural—are used to explain patterns and variations (Christensen et al., 2020).

In this case, the focus is primarily on the relationship between the political executive and the expert bodies subordinate to the ministry in charge of handling the pandemic. But the role of administrative executives in the MH, of whom some also have relevant expertise in the field in question, will also be discussed. The subordinate expert bodies are also administrative bodies in many respects, but the focus is on their output and expertise related to handling the pandemic.

## Theoretical basis

The seminal work of Aberbach et al. (1981) teased out four images of the politics/administration dichotomy. Image I represents the most pure distinction according to which politicians make policy and civil servants administer and implement it. Image II stresses that politicians and bureaucrats both participate in policymaking, whereby politicians advance interests and values, while bureaucrats supply facts and knowledge (cf. Simon, 1957). Image III asserts that both politicians and bureaucrats engage in policymaking, with politicians representing the broad and diffuse interests of the unorganized public, and bureaucrats asserting the narrower interests of organized actors. Image IV is a pure hybrid of the other images. A lot of studies have followed up on this initial study. In a recent survey of local politicians in four countries, Bækgaard et al. (2020a) found that role perceptions are much more complex and seem to vary much more between and within countries than suggested by the Aberbach, Putnam and Rockman (1981) study.

Lodge and Wegrich (2014) discuss four different types of administrative capacity in the modern state, all of which are relevant for analyzing crisis management. The four types are delivery capacity, regulation capacity, coordination capacity, and analytical capacity. Scientization relates to analytical capacity, i.e., the government needs policy advice and information from experts to make decisions, particularly in crisis situations (Moran & Green, 2020). One important aspect of analytical expertise is how it is organized, meaning whether it is close to the political executive, as in agencies, or needs to be acquired from outside the government.

During a pandemic, evidence-based decision-making faces three obstacles (Rubin et al., 2021). First, there is a lack of reliable evidence about what to base decisions on combined with a high public risk of doing nothing. Second, new evidence about the spread and seriousness of the virus and about the effectiveness of the measures taken is continually emerging. This is illustrated by a principled approach versus a pragmatic approach (Boin & Lodge, 2020). Third, there may be a breakdown of the usual hierarchy of scientific evidence in the policymaking process. The dominance of health experts is challenged by advice from experts outside the health sector.

According to a *structural-instrumental perspective*, the overall relationship between political leaders and experts can be of two types: either hierarchical or characterized by heterogeneity and negotiations (March & Olsen, 1983). In a hierarchical model the top political leadership will make the most important decisions in a crisis, and experts will be subordinate, meaning that their knowledge and advice will be used to achieve public goals and balanced against other considerations by the political executive. A negotiation model takes as a point of departure the idea that public organizations are heterogeneous regarding the interests and views of both political and expert actors. Cyert & March (1963) assert that under such conditions, decisions can be made in one of three ways: the majority decides; there is a compromise; or there is a quasi-solution of conflicts, which can be based on sequential attention to goals, in others words, ‘agreeing to disagree’. This means that different actors will have their wishes fulfilled at different points in time or else priority will be granted to local rationality, whereby different subordinate bodies act according to their own goals and values (Allison, 1971). A main argument is that the organization of expert knowledge within the government conditions expert influence (Christensen, 2021).

Here, this perspective is used in three ways: first, to describe and analyze whether the political and administrative executives in the ministry determine the major decision-making premises and take the decisions required to handle the crisis; in this case expert advice will be subordinated to and balanced with broader political and societal considerations. Second, to examine the relevance of the hierarchical organization of the two main expert bodies involved in fighting the pandemic. Third, to address disagreements between the political leadership and the expert bodies as well as between the two expert bodies, and to examine how this plays out regarding major decisions taken on handling the pandemic.

According to a *cultural perspective*, public organizations will develop informal norms and values in a gradual process of adaptation to internal and external pressure, giving them a unique identity (Selznick, 1957). These informal norms will develop with formal structures and norms as major constraints. Cultural identity can be connected to a wider cultural context, but here the focus is on culture in a narrower political-administrative context. In this context culture can be collaborative, political, or expert-oriented; moreover, it does not need to be homogeneous but will follow what is seen as culturally appropriate (Christensen & Røvik, 1999). Cultural traditions and path-dependency are highly significant for understanding public decision-making based on such a perspective.

This perspective is used to analyze whether diverse cultural traditions are significant in the approach taken to combating the pandemic and for the degree of agreement about measures to be taken, or whether the culture is relatively homogeneous in a small country generally characterized by a close relationship between political leaders, administrative leaders, and experts (Christensen, 2003). The relevance of the different cultures of the two expert bodies is also discussed, including the fact that, while they are normally subordinated to the ministry but on a par with each other, during the pandemic the NDH has coordinated with the NIPH and has been superior in the hierarchy.

## Context

The Ministry of Health and Care Services (MH) is the central crisis management ministry in Norway for handling a pandemic, and its subordinate agencies, the Norwegian Directorate of Health (NDH) and the Norwegian Institute of Public Health (NIPH), are the main expert bodies. In addition, the prime minister and the cabinet are central actors.

For this study, analyzing the roles and collaboration between the two expert bodies, including their main tasks and their formal and actual relationship to the ministry, is paramount. The NDH has three main roles: as an executive agency, as a regulatory authority, and as an implementing authority in the area of health policy. In its professional capacity, it has two main tasks related to handling COVID-19—gathering knowledge and experience on health issues and giving advice and guidance on strategies and measures to the ministry. In 2019, the NDH had 545 employees.

The NIPH acts as a national competence institution for infectious disease control, which is most relevant for the pandemic; in this area it has far more resources and expertise than the NDH. It is responsible for knowledge production and systematic reviews of the health sector and provides information about the population’s health, the factors that influence it, and how it can be improved. Key activities are emergency preparedness, investigations, consultancy, laboratory services, and research. As of January 1, 2019, the NIPH had 866 employees.

During crises and pandemics there is a hierarchical relationship between the two agencies. The NDH leads the coordination of the health sector as the superior agency and decides which measures are necessary. The NDH has the authority to impose regulatory measures in pandemics. This means that the NIPH sends its expert advice to the NDH, which then balances this with broader health and other concerns and then forwards its advice to the ministry.

One can distinguish between three types of expert assessment. First, there are the narrower assessments by health professionals, such as analyzing the cause and spread of the pandemic and the costs and health effects of various regulations. Second, there are assessments of other factors such as the losses and challenges for families, employees, and employers posed by the regulations, gaps in schooling for children, the impact of restrictions on leisure activities and cultural events on welfare, and the negative impact of restrictions on individual rights and freedoms. Third, there are broader value assessments, such as how to assess the trade-off between life and health, on the one hand, and individual freedom and economic considerations, on the other. These are the responsibility of the political leadership. The core responsibility of the NIPH is to assess the first set of narrower considerations, while the role of the NHD is primarily related to the second broader set of assessments; the trade-offs between different values and considerations come under the purview of the government. In practice, however, there are grey zones between the responsibilities of the NHD and the NIPH. The NIPH has gone beyond the narrower issues requiring health expertise, while the NHD has also been involved in epidemiological assessments, an overlap that makes their relationship potentially challenging.

## Method

The main data for this paper are provided by public documents, including reports, websites, and media coverage related to the political and expert authorities during the first year of the pandemic. The report from the Enquiry Commission on the authorities’ crisis management of the corona pandemic is also an important source, including transcripts from its interviews with the main actors (NOU 2021:6). The point of departure is the initial and primary stance of the main expert body, the NIPH, on some major issues and the decisions taken by the government to tackle these issues, i.e. its regulations, deregulations and re-regulations, particularly in cases where they disagreed. A high level of transparency and open access to policy advice has made it easy to access the diverging views of expert bodies. The advantage of this approach is that it allows us to contrast expert advice and political decisions in cases where they differ. The disadvantage is that it could lead to an exaggeration of the degree of conflict. Moreover, on some issues the government and NIPH may have changed their views in complex ways.

The focus on the NDH is secondary, partly owing to more limited access to data. While the NDH has published reports on some issues, on others it is difficult to find out what its stance is, since this is communicated confidentially to the government. Thus, information is overall more limited. One would assume that the relationship between the ministry and the NDH would involve the NDH giving the ministry advice based on the NIPH’s views and its own expertise, but it may also include actions based on anticipated reactions from the government and pressure from the political executive to filter the NIPH’s advice in a certain way and thus exert further political pressure on the NIPH.

## Infection control measures—disagreements between the political leadership and the main expert bodies

*Ministry-agency relations.* According to the Enquiry Commission, the division of responsibility between the Ministry of Health and Care Services (MH) and the subordinate agencies has been rather unclear during the management of the COVID-19 crisis, especially with respect to the Norwegian Directorate of Health (NOU 2021:6). Overall, the ministry has exerted rather tight control and its secretary general has played an active role. Altogether the NDH and NIPH have received more than 300 written assignments from the ministry during the pandemic of which several were rather small, overlapping, and with short deadlines. Thus, the ministry’s crisis management and coordination has been rather detailed, constraining its ability to make holistic and strategic assessments. However, the ministry has also delegated crucial responsibility to the NDH, such as the power to ‘push the big red button’ on March 12, 2020, with the introduction of draconian measures (Høie, 2021; Solberg, 2021). Later, daily collaborative meetings between the MH, the NDH, the NIPH, headed by the secretary general in the MH were an important ad hoc coordinating mechanism and they were also allowed to participate in the regular cabinet meetings and the inner cabinet covid-19 committee.

*The relationship between the NDH and the NIPH*. In the first phase of the pandemic, when the agencies were under great pressure and major decisions had to be taken quickly, there were cooperation problems and disagreement between the NIPH and the NDH about the distribution of roles and tasks and about who was responsible for what, which according to the NIPH resulted in confusion and extra work (NOU 2021:6). In early March 2020, the NIPH wanted to wait until May/June before introducing major infection containment measures, by which time the spread of infection would have been more advanced. Until March 6, the NIPH and the NDH held separate press briefings, which started to become a problem. A crisis meeting between the two agencies was held March 8. The situation then changed quickly. On March 10, the minister of health and care gave a briefing in Parliament but did not mention that the country could close down within a few days. Later the same day there was a meeting in the Emergency Response Committee for Biological Issues headed by the NDH and attended by the NIPH. At this meeting, the NIPH suggested various measures. It warned against starting too early with contact-reducing measures and was worried about setting the limit on public gatherings too low. The decision to close down Norway was, however, taken at a meeting on March 12, which the NIPH did not attend, and it was later given fifteen minutes to assess the measures proposed by NDH (NOU 2021:6). The government had given the director of the NDH the discretion to propose major regulations without involving the NIPH, which the director of the NIPH later said was rather problematic. The secretary general in the MH indicated that this was appropriate, since he thought that the NIPH should not play an active role in these regulatory decisions (Larsen, 2021); this was a controversial decision.

Once the COVID-19 regulation was introduced, the main procedure was that the NIPH’s policy advice was conveyed through the NDH as an advisory body to the political executive. Owing to internal challenges with unclear roles and responsibilities, the minister of health sent a letter in November 2020 to all involved bodies, informing them about the roles and responsibilities of the NDH. He said the NDH should coordinate, but it had no power to steer the other bodies. In case of disagreement, the authorities should try to find an agreement. If they were not able to agree, the ministry would have to decide how to resolve the disagreement. Thus, during the first phase of the pandemic, collaboration between the NDH and the NIPH was difficult, and coordination was less than optimal, but these problems decreased somewhat over time.

One rather systematic and interesting feature during the pandemic has been the general attitudes to the coupling between evidence-based knowledge represented by the NIPH and the regulations, which were a main responsibility of the NDH. The NIPH served mainly as a scientific epidemiological advice body while the NDH was a bureaucratic health authority, but in practice these roles overlapped. The government, including the NDH, has argued that the knowledge basis for the regulatory measures was weak, but that urgency based on the precautionary principle and a suppression strategy drove a lot of the regulations. The advantage of this strategy is that the government has been transparent, which is natural in such a massive health crisis, but can also be seen as a political precaution. The minister of health said in an interview with the Corona Commission that it was better to be criticized for doing too much than too little (Høie, 2021). The disadvantage is that existing knowledge has been downplayed and regulations have become very complex, because new regulations and periodic deregulations and re-regulations have been added, in a layering process (Mahoney & Thelen, 2010). The NIPH has taken a more cautious attitude, more informed by the proportional principle (Boin & Lodge, 2021). It has talked more in terms of what is known or not known and stressed that trying to find out the effects of specific regulatory measures is important.

*Overall pattern.* The authorities were not well prepared for the COVID-19 pandemic, even though the pandemic was an anticipated crisis with a high risk of occurring. The analytical capacity of NIPH and NDH was rather poor. Emergency plans had been conceived for a flu-like pandemic and there was a lack of training and no plans for a (semi)-lockdown. The expert bodies had trouble making sense of the development of the pandemic in the first phase and in forecasting how infections would develop.

During most of the pandemic, the political leadership and the NIPH seem to have disagreed on many questions, even though they sought to present a united front towards the public, which may reflect political pressure on the NIPH. On most central questions, the NDH agreed more with the government than with the NIPH, which reflects its proximity to the political executive. This pattern began in the first wave in March 2020, when the main rhetoric of the government was about stopping the pandemic; the NDH, by contrast, used the concept of suppressing the virus, while the NIPH focused on ‘slowing down’ the spread of infection. From then on, the NIPH argued for fewer restrictions and more liberal policies, called ‘evidence-based’ by some, but this more liberal line of argument did not have much impact on the government’s actual pandemic regulations.

In late October 2020, at the start of the second wave, some of the same pattern occurred. The government anticipated a new wave and thought it would be severe. The NDH supported this position, while the NIPH defined the situation as more uncertain. In the third wave, which started in mid-February 2021, fewer disagreements were revealed, even though the regulations were stricter than ever. There could be at least three reasons for this. The first is that the two expert bodies were indeed more in agreement during the third wave, having engaged in a kind of mutual adaptation. A second reason could be that some conflicts now took place internally rather than in public. A third possibility is that the longer the pandemic lasted, the more problematic it became to advance some of the NIPH’s views, even though they can be seen as evidence-based. An examination of individual issues reveals, however, that the pattern is still mixed concerning ‘who is right’ and there is also still a lot of ambiguity and uncertainty about which regulations have been effective.

*Attitudes to specific regulatory issues.* Travel restrictions were one of the first areas of conflict and disagreement to be examined in more detail. Regarding *international travel*, up to the end of February 2020, the NIPH did not recommend any major travel restrictions. It was possible for Norwegians to travel abroad without any testing or quarantine when they returned. When the travel and border restrictions were imposed in March 2020, they were rather weak, but still involved some limits on travel, especially for foreign citizens. The NIPH was rather skeptical towards this regulation, and one of its main arguments was that the virus had already entered Norway, mainly through ski tourists coming home from Austria and Italy, and that more border restrictions would be less effective than fighting the virus inside Norway.

Later, the NIPH moved in a more restrictive direction on this question. In June 2020, the political executives in the ministry and government opened the borders for foreign guest workers and granted them an exemption from the ten-day mandatory quarantine, against the advice of the NIPH and the NDH, who warned that there was a particularly high risk that this group would import infection. This exemption was introduced after lobbying from industry and the trade unions. Later, in the second wave from late October 2020 and the third wave from mid-February 2021, the NIPH recommended harsher regulations. At the beginning of 2021, the borders were in fact almost completely closed, even for foreign guest workers, to avoid importing infections, which resulted in a public debate and later a few adjustments for health workers and eventually for other groups as well.

Regarding *internal travel*, the government decided during the first wave to forbid people to go to their second homes, i.e. cottages up in the mountains or summer houses, a restriction that lasted for five weeks, including the Easter of 2020. This has never been repeated, even though some municipalities tried to introduce weaker regulations of this kind during the third wave. Norway was one of the very few countries that introduced such a regulation. It was the result of some proactive mayors convincing the government that this was a good idea. The main argument was that people from the epicenter(s) would bring the virus to smaller communities in the countryside and completely overwhelm the capacity of the local health system. The NIPH was against such a regulation, arguing that it would not stop the spread of the virus. Therefore, it did not see such a regulation as necessary or realistic. The government and the NDH never admitted that this had been a mistake, but it was very clear before the Easter of 2021 that this regulation would never be repeated.

It was decided in March 2021 to *close all kindergartens and schools* for five to six weeks. The government’s argument, supported by the NDH, was that children and young people were also contributing to spreading the virus, since they were in close proximity in these institutions. The NIPH was initially strongly against this regulation, especially for kindergartens and elementary schools, for two reasons. First, it thought that children played a marginal role in the pandemic and that the social consequences would be too negative, especially for vulnerable children. The latter argument was repeated later by the NIPH, when it was more eager than the government to open the schools again in late April/early May 2020. It is interesting that the NIPH’s concern for vulnerable young people, supported by a report from an expert group, never figured prominently in the government regulations, i.e. it was not prioritized relative to health and economic concerns. In retrospect, the prime minister admitted that school closures were one of the early measures that she regretted, and the Corona Commission was also critical on this point (NOU 2021:6).

The regulations with respect to schools were rather complex and they aroused the skepticism of the NIPH, which pointed to the difficulties of children having on-line school at home while parents were also having to work from home. In the second wave, a traffic light model was established whereby in municipalities with high infection rates, schools should not be closed but should adhere to red level regulations, including strict contact-reducing measures and less frequent school attendance. This system helped to keep schools open, but it also fluctuated a lot and was difficult to understand. This model was combined with a complicated system of geographical transmission zones, with various and changing regulations. During the past year of the pandemic, the government has never used a national regulation on closing kindergartens and elementary schools nationwide again, but it has done so in specific areas at various times. On March 15, 2021, the city of Oslo decided to close schools and kindergartens for ten days against the advice of the NIPH.

A fourth issue of disagreement was the use of *face masks*, which Norway introduced rather late compared with other countries. The first regulation on this came in mid-August 2020 in Oslo and some nearby municipalities where infections were increasing, but even then the use of masks was only recommended in situations where people were not able to keep at least one meter distance. This resulted in rather few people using masks, for example, on public transport. This changed as the second wave developed from late October. Later it became a national recommendation, although in some municipalities it is mandatory, and in reality, nearly everyone started to wear masks in stores and on public transport.

What is interesting is that the NIPH was skeptical about masks for a long time. It repeatedly cited international research showing that wearing masks had little effect, something that critics said was plainly wrong. They even gave an example, repeatedly saying that 200,000 people wearing masks would potentially save one life. NIPH also argued that masks could be harmful and that they were often used in the wrong way. Despite all this, the government decided to introduce the mask regulation, without much scientific supporting evidence, probably mainly because the measure had been widely adopted in other countries. Eventually the NIPH supported it but argued that the reason for its change of stance was the higher number of transmissions. In a publicly broadcast debate, the minister of health reacted strongly when the mediator brought up this difference in opinion between the government and the NIPH, indicating that the NIPH had been put under a lot of political pressure on this question.

## Discussion

*Balancing different values and concerns*. Four main considerations have been addressed in the management of the pandemic in Norway—health, the economy, social considerations, and civil rights—of which health has been most important for the government with the economy second, while health and social concerns were most important for the expert bodies (Christensen & Lægreid, forthcoming). With respect to health, the government sought to suppress or stop the spread of the virus using a precautionary principle, in order to avoid overloading the healthcare system with severe cases requiring hospitalization. But as one of the leaders of NIPH said early in the pandemic, the precautionary principle is potentially ambiguous and does not really focus preventive efforts in a very specific direction. This implies that the government created a rather broad discretionary space for itself in its handling of the pandemic and used that space to listen more to the NDH than the NIPH.

The social consequences of the measures received some attention, but much less than other concerns (Expert group, 2020). Protecting elderly people was prioritized, but still many old people in nursing homes died of the disease. Vulnerable groups among children and young people were prioritized after educational institutions and sport and leisure activities were closed down, but despite this they lost most of their normal safe environment as a result of these measures. However, addressing this concern did not receive much support, even though it was evident that children and young people with special needs had had a difficult time because for various reasons their special services had basically disappeared during the pandemic (Christensen, 2021; Expert group, 2020). While the concerns of this group were part of politicians’ and administrative leaders’ rhetoric, in practice they were not accorded a high priority.

Individual and civil rights likewise tended to be consigned to the background during the management of the crisis, especially during the first phase of the pandemic (NOU 2021:6). The government did not pay much attention to the question of whether the infection control measures were in line with the Constitution and human rights. Some leading law professors, however, took these questions very seriously (Graver, 2020) and criticized both the passing of the exemption law and how municipalities had used the Infection Control Act to introduce strict local regulations.

Overall, these difficult trade-offs between different values and goals were not delegated to experts alone, but were handled by political executives informed by expert advice (Christensen & Lægreid, 2020b). In fact, there was rather tight control and instruction from the parent ministry—a centralized approach that is rather common in the management of major crises (Boin & ‘t Hart, 2003). The main pattern was governance informed by experts not governance by experts.

Paradoxically, the NIPH as an expert body with supposedly narrower attitudes than the MH and the NDH used rather broad arguments with respect to the various regulations. First, during both the first and second waves, the NIPH argued broadly against the strict regulations (Christensen & Lægreid, 2020b). It emphasized that they should be more evidence-based and it expressed concern about the human rights implications of limiting movement. It was also concerned about the effects on employment and about the social effects of closures that left vulnerable children isolated in their home environment without the usual social services. They were also critical of the restrictions on freedom of movement implied by the ‘cottage law’ (Christensen & Lægreid, 2020a). The closure of kindergartens and schools gave rise to many arguments regarding social vulnerability, while the closure of borders and the imposition of quarantine again raised questions about their infringement of people’s rights and about whether these measures were evidence-based. Finally, with respect to face masks it pointed to the lack of scientific evidence and the risk of using them wrongly. Thus, the NIPH used a broad range of arguments to criticize the measures, whereas the views of the government and the NDH were much narrower, which may seem paradoxical.

A survey of the NIPH’s basic attitudes to central regulatory questions over the past year, especially seen through the lens of the Corona Commission, yields a rather mixed picture when compared with those of the MH and the NDH. While its initially liberal attitude to border control seems to have been proven wrong, its much more restrictive attitude to migrant workers and its criticism of allowing them to enter the country unchecked seems to have been borne out. Second, its resistance to kindergarten and school closures and its insistence that this issue should be considered from a broader perspective has received much support in retrospect, including from the government and the NDH, which later became self-critical of these measures. Third, the NIPH was probably right to resist the cottage law, especially since it was never repeated. NIPH resistance to face masks, on the other hand, is more difficult to judge.

*Revisiting the theoretical perspectives.* Going back to the two perspectives outlined initially, one can use them in various ways to interpret the main results. First, the structural-instrumental perspective digs into the relevance of the formal structure of government (Egeberg & Trondal, 2018). The management of the crisis strengthened the power of central government, especially the Cabinet, the PM and the Ministry of Health and its subordinate agencies (Christensen et al., 2016). The hierarchy was strong, and the ministry exerted rather tight control. Where the expert bodies are formally situated would seem to have been of major importance for the approach to handling the pandemic (cf. Egeberg, 2012). Because of its structural proximity to the political leadership, the NDH, seems to have been delegated a lot of authority as a coordinative body and it systematically sided with the government. The NDH had great strategic power, because it was a go-between body, filtering the NIPH’s views and transmitting steering signals and regulations from the political executive. The NIPH had more autonomy from the political leadership compared with the NDH, but also less access to and influence over it because of its subordinate structural position in the crisis management process (Christensen & Lægreid, 2020a). It was also seen as less important by the administrative leadership in the MH. In other words, the body with the greatest expertise regarding the cause and effects of the pandemic did not have the greatest influence on the handling of the pandemic, which is rather paradoxical.

The structural perspective can also provide some insight into the complexity of the regulations, which were composed of formal rules and advice. Given the uncertainty of means-end knowledge regarding the effects of the various regulatory measures, as well as uncertainty regarding the spread and severity of the virus, a pragmatic strategy may make sense, adjusting the means and measures as new information emerged during the pandemic (Boin & Lodge, 2020). While the government had regulative capacity during the pandemic, its pragmatic approach also resulted in a rather complex system, which the regulators, the expert bodies, and especially the public had problems understanding. The national COVID-19 regulations have changed frequently during the pandemic, and this complexity has been compounded by changes in local regulations. The impact of national and international learning was not very evident in this regulatory approach to the pandemic, which was a weakness.

Referring to Lodge & Wegrich’s (2014) types of capacity, one can say that regulatory capacity was used to handle the pandemic, albeit very proactively, based on a precautionary principle, making for a great deal of complexity. This created problems for both coordinative and delivery capacity because other actors found it difficult to follow the government’s lead. Finally, analytical capacity, represented primarily by the NIPH and partly by the NDH, was low in the first phase of the pandemic and later consigned to the background. Overall, the management of the crisis scored high on social and political control through centralization of power, which fits the structural perspective well, but it scored rather low on means-end knowledge regarding the effects of different regulatory measures (cf. Dahl & Lindblom, 1953). However, over time it also allowed for pragmatic adaptation of regulatory measures based on experience.

The *cultural perspective* provides insight into the relevance of political-administrative traditions and path dependency. Despite some coordination problems, differing expert advice, and tight control from the ministry, much of the collaboration between the ministry and the subordinate agencies and between the agencies worked relatively well and displayed the flexibility typical of the Norwegian collaborative decision-making style. Although Norwegian political-administrative cultural identity is traditionally homogeneous (Christensen, 2003), the handling of the pandemic highlighted the importance of cultural variety and heterogeneity. While the political and administrative leadership has more discretion in times of crisis (cf. Thompson, 1967), it was also under strong pressure to balance all the different concerns and decision-making premises and reach decisions quickly under conditions of great uncertainty and grave consequences (Boin et al., 2020). To accomplish this balancing act, it consulted expert bodies, parliament, and interest groups, following the logic of appropriateness in the consensus- and trust-based collaborative Norwegian tradition (cf. March & Olsen, 1989). This cultural path led it to adopt the precautionary or ‘better safe than sorry’ principle, the logic being that doing too little could lead to a political disaster while doing too much might earn it criticism that would be easier to handle, which seems to have been the case (Høie, 2021).

The NDH’s cultural path could be characterized as experiencing cultural cross-pressure between serving the political executive and filtering and passing on advice from the NIPH (Christensen & Lægreid, 2020a). In a unique situation like this pandemic and with additional authority from the government, it adjusted its cultural path in favor of the concerns of the political leadership; the strong representation of social scientists among its staff likewise furthered this approach. The basic cultural role of the NIPH is that of the most typical expert body, with the broadest set of specialists relevant to the pandemic and the closest ties with international expert bodies and relevant research communities. This path was more ‘theoretical’ and removed from the realities facing the political executive, making it easier to adopt a strategy informed by the proportional principle in handling the pandemic (Boin & Lodge, 2021). The NIPH, with its homogeneous demographic profile, was inclined to recommend caution, look for evidence, and advise the government to take one step at a time, which partly explains its lack of influence on policy.

*The politics-administration dichotomy*. Going back to Aberbach et al. (1981)—how do the data compare with the images outlined in that study? What one see is a mixed order in which the role perceptions are much more complex when the policymaking process plays out as cooperation between the political executive, administrative leaders, and expert bodies (Olsen, 2010). The expert bodies are not only technical and neutral advisors but play an important political role in the policymaking process, especially the NDH with its delegated authority and structural proximity to the political leadership. While politicians pay heed to stakeholder interests and underlying values, the experts supply facts and knowledge, as outlined in Simon’s (1957) basic insights. But in the crisis management in question here, the NDH has been rather well integrated into the political leadership and has both furthered and represented its norms and values (cf. Boin & Lodge, 2020). The NIPH’s advice, on the other hand, is based primarily on professional norms and values. Apart from the pure scientific advice from epidemiologists and virologists, the institute has repeatedly warned about the negative effects of the pandemic measures on vulnerable young people and families working from home, which implies a broader viewpoint.

A main lesson from this study might be that a mixed decision-making regime could be appropriate in the management of a pandemic, going beyond the narrow expertise of epidemiologists. This means that when the existing knowledge base is uncertain, such knowledge is used more to inform decisions than to drive them. Norway seems to have come close to such a strategy. It did not delegate responsibility purely to experts, as Sweden did (Pierre, 2020), and even if the government implemented more radical measures than recommended by some health experts, these decisions were taken in close collaboration with the health authorities and communicated in a transparent way (Christensen et al., 2020b; Rubin et al., 2021). Denmark’s policymaking in the initial phase was characterized by more centralization, led by the PM, but also by miscommunication between the government and the health authorities (Bækgaard et al., 2020b). Norway was also quick to diversify its list of experts informing the decision-making process, bringing economic experts and experts on the effects of school closures into its team of advisers.

## Conclusions

Going back to the research question, the conclusion is that the experts and the political leadership disagreed on some of the regulatory tools to fight the COVID-19 pandemic. On several issues, the political executive decided on more radical measures than advised by the experts. There were also some disagreements among experts. Overall, the Norwegian Institute of Public Health recommended softer measures than the Norwegian Directorate of Health did. As one might expect, both structure and culture contribute to explaining this pattern. A main topic for future research would be to analyze responses on the same research questions in other countries.

What can this study say about the relationship between politics, administration, and expertise? First, managing a crisis like the COVID-19 pandemic often leads to more centralization of power in the hands of the political leadership (Boin & Lodge, 2020). This was the case in Norway. In addition, central administrative leaders, and to some extent experts in central agencies, increased their power relative to lower levels, which happens more often in a unitary state (Christensen, 2003). The politicians took a hands-on approach, with the policymaking process informed by expert advice rather than being decided on these expert premises. Rather than a dichotomy between the political executive and the administration, one see a blurring of the borders and significant grey zones. However, the significant differences between expert advice and political decisions, as well as the varying opinions of the experts themselves, were rather open and transparent, and the main actors freely admitted that important decisions were being taken under conditions of great uncertainty and urgency (Boin et al., 2020).

Second, in handling a pandemic in modern times, centralization also means that a blurring of the dichotomy is part of the crisis communication strategy (Christensen & Lægreid, 2020b). At the beginning of the pandemic, the Norwegian political leadership held daily press conferences with the leaders of the subordinate expert bodies, which in some ways made these leaders part of the government’s communication strategy. Such a strategy was intended to make sense of the situation, to calm people down, and urge them to follow the rules and advice, but of course also to enhance the legitimacy of the political leaders by casting them as rational and pro-active (Boin & Lodge, 2020). In this process, there was little room for open disagreement between politicians and experts, or among experts, since the motto was ‘we must stand together’. This feature subsided in the third wave of the pandemic, when the handling of the pandemic was reviewed, and the media became more aggressive in revealing disagreements.

Third, even though the handling of the pandemic was more centralized than other public decision-making processes, it in many ways also reflected the underlying cultural appropriateness, defined in the Norwegian context as the peaceful integration of different actors in public decision-making (Christensen, 2003; Olsen, 2010). The importance of following appropriate processes and procedures was underlined, and experts were consulted as were the parliament and diverse interest groups. This integrated decision-making style coincides more closely with images II and III than with a sharp division between politics and administration.

Fourth, there was little about the handling of the pandemic in Norway to indicate that there was a scientization of politics or that experts are about to colonize politics, as many have interpreted the experience in Sweden (Petridou, 2020; Pierre, 2020). Quite the contrary: it begs the question of whether this centralized handling might have a lasting effect on governmental decision-making processes after the pandemic. There are, however, several features that do not point in this direction. One is the growing complexity of politics and ‘wicked issues’, making politicians more dependent on the experts, whether national or transnational, for example through the EU. Another related one is the growing dominance of experts in public committees in Norway (Christensen, 2018). A third is the continued support for the tripartite collaboration in the system.

## Declarations

Not applicable (None).
